# The Effects of Mild Traumatic Brain Injury on Cognitive-Motor Integration for Skilled Performance

**DOI:** 10.3389/fneur.2020.541630

**Published:** 2020-09-16

**Authors:** Lauren E. Sergio, Diana J. Gorbet, Meaghan S. Adams, Danielle M. Dobney

**Affiliations:** ^1^School of Kinesiology and Health Science, York University, Toronto, ON, Canada; ^2^Centre for Vision Research, York University, Toronto, ON, Canada; ^3^Vision-Science to Application (VISTA) Program, York University, Toronto, ON, Canada; ^4^Toronto Rehabilitation Institute, University Health Network, Toronto, ON, Canada

**Keywords:** movement, cognition, eye-hand coordination, sensorimotor integration, concussion, blast exposure, neuroimaging, motor psychophysics

## Abstract

Adults exposed to blast and blunt impact often experience mild traumatic brain injury, affecting neural functions related to sensory, cognitive, and motor function. In this perspective article, we will review the effects of impact and blast exposure on functional performance that requires the integration of these sensory, cognitive, and motor control systems. We describe cognitive-motor integration and how it relates to successfully navigating skilled activities crucial for work, duty, sport, and even daily life. We review our research on the behavioral effects of traumatic impact and blast exposure on cognitive-motor integration in both younger and older adults, and the neural networks that are involved in these types of skills. Overall, we have observed impairments in rule-based skilled performance as a function of both physical impact and blast exposure. The extent of these impairments depended on the age at injury and the sex of the individual. It appears, however, that cognitive-motor integration deficits can be mitigated by the level of skill expertise of the affected individual, suggesting that such experience imparts resiliency in the brain networks that underly the control of complex visuomotor performance. Finally, we discuss the next steps needed to comprehensively understand the impact of trauma and blast exposure on functional movement control.

## A Complex Skill Requires a Complex Brain: An Introduction to the Neural Control of Rule-Based Skilled Performance

### Imagine

At work, you operate a drone aircraft. You have one joystick that sends the drone up or down and turns it in a circle, and another that flies it forward, back, or side to side, and all the while you look at a computer monitor showing images from the drone's camera to allow you to direct its movement. On break you enjoy a cup of tea, reaching your arm forward to pick up your teacup.

Operating the drone and reaching for the cup are examples of visually-guided arm movement that vary widely in their complexity. How are you able to perform these tasks proficiently and relatively effortlessly? Reaching for and interacting with objects in the environment is a common daily task, but one that can vary widely depending on task conditions. Looking at and then reaching for a teacup is an example of a direct interaction. The visual stimulus guiding the action is itself the target of the action. In contrast, remotely operating a drone is an example of an indirect interaction: there is an intermediary between the action and the target. Direct interactions are guided by standard sensorimotor mappings within the brain. Indirect interactions are guided by non-standard sensorimotor mapping, and rely on different neural computations that must incorporate the spatial dissociation of gaze, attention, and overt motor output (1).

“Transformational” non-standard mappings use specific spatial algorithms to relate the position of the visual cue to the direction of action, such as relating horizontal mouse movement to vertical cursor movement. The different levels of visuomotor compatibility in transformational mappings are achieved by two sensorimotor strategies: sensorimotor recalibration and visuomotor adaptation. Sensorimotor recalibration allows for adaptation to spatial orientation differences by remapping between two sensory modalities (2–4). Because this is an implicit recalibration, there are after-effects if the source of recalibration is removed. Think of the lightness you feel for the first few steps after taking a long trek with a heavy backpack. In comparison, visuomotor adaptation, or “strategic control” (5) relies on a mental rotation to align the required limb movement to the spatial location of a target. It is more explicit, does not produce after-effects, and can require rule integration. Note that these terms are not used consistently across the motor adaptation literature [for comprehensive reviews see (6, 7)]. Lastly, in order to execute skilled movements smoothly and accurately, the brain must often account for various rule-based situations. Combining thought and action is a process known as cognitive-motor integration (CMI). CMI is essential for the completion of skilled activities that involve non-standard mappings and other complex skills, making it crucial for work, duty, sport, and daily life. While healthy adults perform CMI with little conscious awareness, these skills are not innate and can be affected by brain injury. In this paper, we review three related lines of research: sport-related head impact and motor behavior, blast-related impact and motor behavior, and functional brain neurophysiology and motor behavior. The linking theme is the neural control of rule-based sensory-guided movement in health and following mild brain injury.

## A Failure to Communicate: The Effect of Mild Brain Injury on Cognitive-Motor Integration Behavior

While some acquired brain injury can impose distinct focal damage to specific brain regions, it is a more diffuse injury that typically arises from blast and concussive impact (8, 9). The acceleration and deceleration forces of concussive trauma initiate a “neurometabolic” cascade. This cascade invokes a state of energy crisis from both an increased glucose requirement—needed to restore ion homeostasis—and a decreased supply of glucose through reduced cerebral blood flow (10, 11). Further, axonal injury occurs due to the mechanical shearing and tensile strain from acceleration, deceleration, and rotational forces (12). Such forces lead to altered axonal membrane permeability and ionic disruption, as well as cytoskeletal breakdown (10, 11), all of which can impair neurotransmission. The time frame, however, for the recovery from these effects and their exact relationship to concussion symptoms is not fully understood.

**Figure 1:**
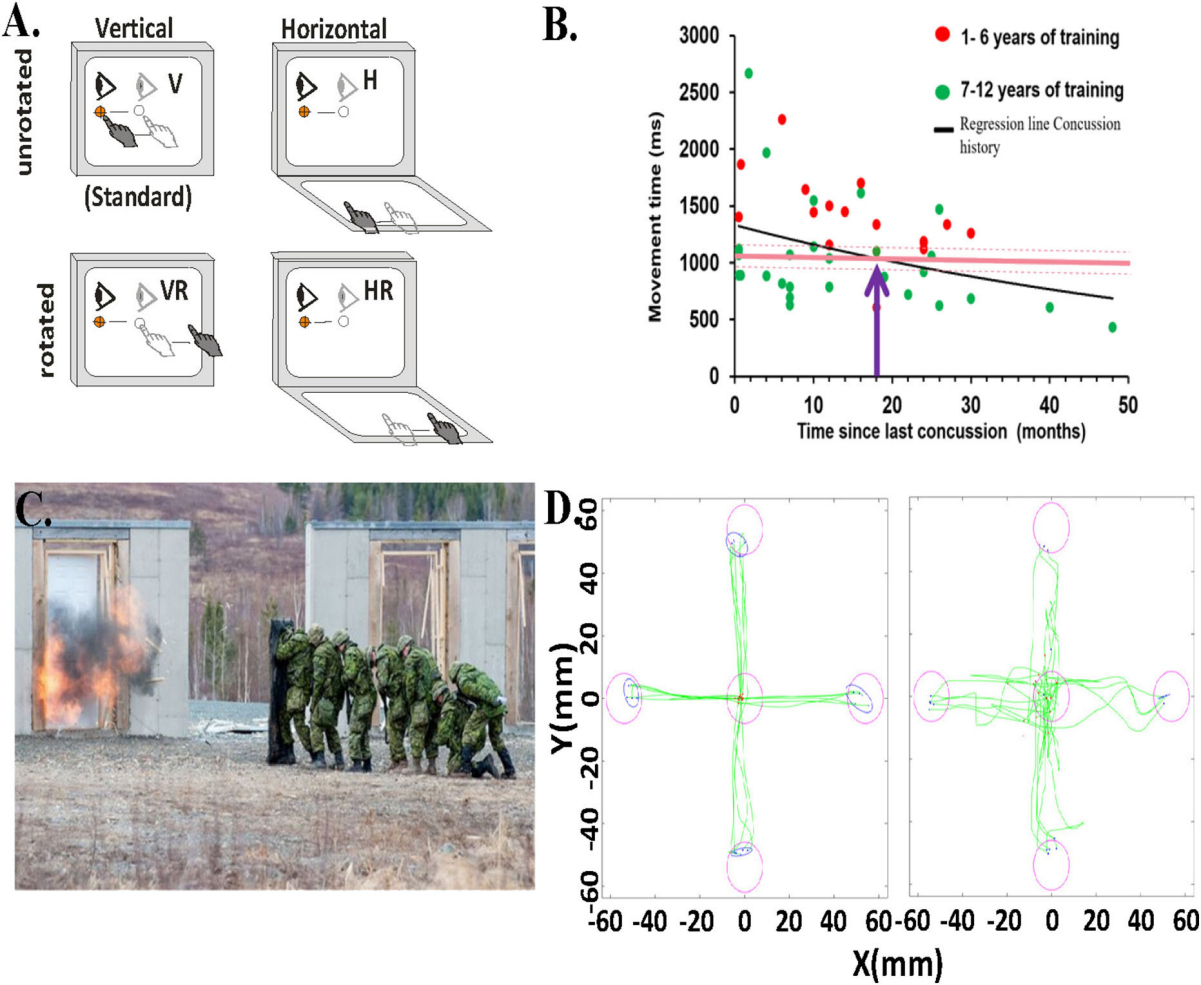
Behavioral task and data. **(A)** Schematic of experimental conditions. Visual stimuli were presented on a vertical touchscreen. Motion was recorded on either that touch screen or a second one placed horizontally in front of the participant. Light gray eye and hand symbols indicate the start position. The dark gray eye and hand symbols depict the movement from start position toward the target. The target was presented in one of four locations (right, left, up, or down). H, horizontally placed touchscreen; V, vertically placed touch screen; R, rotated (180^o^) visual feedback. **(B)** Relationship between the total movement time in the CMI condition and time since last concussion for each child with concussion history and asymptomatic (dots), represented by the regression line (black line). Red dots are those children with 1–6 years of training, green dots are those with 7 or more years of sport experience. Also included is the mean total movement time of the CMI condition of the healthy children (8–16 years old) with no concussion-history (dark pink horizontal line). The light pink dotted lines indicate the upper and lower confidence intervals of the control group's total movement time. The purple arrow denotes where the two lines of the concussion history and no-history controls cross. **(C)** Canadian Forces School of Military Engineering personnel participating in breaching exercises. Photo courtesy of Haley Voutour, 5th Canadian Division Support Group. **(D)** Examples of hand movement trajectories (green) in the Standard (direct interaction) and in the Plane Change+Feedback Reversal condition (“horizontal rotated”) from one control participant (left panel) and from one breacher participant (right panel).

We propose that widespread injury can reduce rule-based skilled performance through a failure to communicate between brain networks. Cognitive brain network alterations have been observed following concussion (13–17). To assess the communication between brain areas for movement control, our group developed a touch screen based functional assessment tool using a visuomotor task that incorporates two forms of non-standard mapping: explicit rule integration (strategic control), and implicit spatial vision-to-hand-motion realignment (sensorimotor recalibration). Portable software applications to assess brain function following injury are increasingly being developed, and offer a useful, pragmatic approach to providing information to the user and their caregivers (18–22). In our task, a basic standard mapping condition has the participant place their finger over a central start target on a vertical touch screen, immediately slide their finger on that same screen directly toward a target that appears, and hold it there. There is universal agreement among the hundreds of youth who have done this task that “it is the most boring video game ever.” It does get more interesting for them, however. In the remaining three cognitive-motor integration (CMI) conditions, one or both forms of non-standard mapping are introduced. In the first CMI condition, the target and cursor are viewed on the vertical touch screen, but the motion of the cursor is reversed from the motion of the finger on that screen. We often see participants talking themselves through this condition (“target is down…slide up slide up!”), suggesting that we are indeed tapping into explicit rule integration associated with this mental rotation. In the second CMI condition, the touch screen recording finger movement is now laid flat in front of them, and the targets are still presented in the same vertical location as before (on a tablet or monitor). This condition requires a more implicit sensorimotor recalibration since the guiding visual information and required motor goal are in different spatial locations. Those who have grown up with computers and video games barely register this condition as a challenge. To this blasé tech-savvy group, however, we throw our third CMI condition, a combination of the two other CMI conditions: one must now think their way through moving opposite to an extrafoveal target (Figure 1A). There is, without fail, a reaction of surprise to this condition. Try it yourself now if you are at a computer with a mouse! Physically turn your mouse around, grab it with the bottom end pointing out, and move your cursor to an icon on your screen. Congratulations, you have just performed cognitive-motor integration using two forms of non-standard mapping. Continue reading to find out what your brain just accomplished.

### How Does Concussion History Influence CMI Performance? Are They Really “Recovered”?

There is evidence that individuals with concussion—a form of mild traumatic brain injury (mTBI)—have reduced capacity for executive function, slower cognitive processing speeds, and attention deficits (23). These data suggest that task switching, required when performing two tasks within the *same* domain (“dual-tasking”), will be more difficult for those with concussion. Concurrent task paradigms using both task switching and integration tasks (combining behaviors from two *different* domains) have been proposed as a means to assess concussion recovery. For example, there are subtle changes in oculomotor function, gait, and balance after concussion, which become more pronounced during both dual-task and integration paradigms (23, 24). Determining if an individual has recovered from concussion is a challenge from a risk management perspective. Clinicians understand the risk of returning athletes too soon to sport and yet the tools used to determine recovery are inadequate, testing one domain at a time (e.g., motor, cognition) or relying on self-report (25, 26). Thus, many individuals are returned to work and sport with impairments that may leave them vulnerable to further injury (27, 28).

We have used our task in several sport concussion studies (29–32), and always observe significant differences in performance outcomes related to both timing and accuracy in concussed athletes vs. controls. We compared youth athletes (8–16 years old) with a history of concussion who were asymptomatic and deemed recovered (33) to age-matched controls with no history of concussion (30). There were no differences between groups on the simple, standard task. In contrast, in the non-standard task, youth who reported a previous concussion were significantly slower in moving from the start to their first stopping point (“movement time,” or MT), which was often before they got to the target. They were also slower in moving from the start to the end targets, a movement which comprised the initial ballistic MT plus the post-pause corrective movement to get to the final target (30). This finding indicates that concussion history is associated with slower non-standard skilled performance in these children, compared to their non-concussed peers. This finding was replicated in a similar cohort of youth athletes who also showed decreased accuracy and increased path variability (31). The significant differences we found in the group with a previous concussion did not subside and return to the level of the matched control group until on average 18 months post-concussion (Figure 1B); that's nearly two seasons later! This finding is concerning given that most youth will have returned to activity within this time frame and thus are likely more vulnerable to re-injury, concussive, or otherwise.

When we examined concussion history effects on CMI performance at the elite level, we found more subtle differences (32). In a study of 102 National Hockey League (NHL) draft prospects (mean age = 17 years, all asymptomatic) we observed that previously concussed participants had significantly decreased reaction times (RT) on the non-standard task compared to the control group, indicating that they were slower to respond to the peripheral stimulus when it appeared (32). Given the dramatic increase in speed of the game between recreational-level and NHL-caliber athletes, we suggest that even minor changes in RT to a given stimulus may represent both a hazard to player safety and an influence on player performance. Noteworthy though are the attenuated effects of concussion on CMI in these elite vs. the non-elite young athletes in our other studies. Experience in complex tasks may be linked to a neurological “reserve” that appears to compensate for behavioral performance (32). The concept of “reserve” is one that is more commonly used with respect to cognition (34, 35), posited to provide a protective effect against cognitive decline associated with aging or disease. One can think of enhanced skilled performance through years of training as offering this same level of protection against performance decline following brain injury, via more efficient and resilient brain networks needed in the control of complex skills.

### What About Low-Level Blast Exposure and Cognitive-Motor Integration Performance?

In a recent study in collaboration with Development and Research Defense Canada, we examined the impact, literally, of repeated blast-wave exposure over a period of years. We did this by looking at the CMI performance of 19 breaching instructors (those who teach others how to forcefully open closed/locked entryways) and range staff having many years of service, which came with many years of exposure to low level blast explosions (Figure 1C). We compared their performance to that of 19 age- and sex-matched Canadian Armed Forces non-breacher control participants. Similar to what we observed in experienced athletes, our preliminary results show a significantly greater variability in reaction time (*p* < 0.05, equality of variance test) in the most challenging CMI condition in the breacher group (36). We also observed slower reaction times and worse accuracy in the standard (Cohen's D: 1.11, *p* < 0.01) and horizontal (Cohen's D: 0.94, *p* < 0.01) visuomotor conditions in the breacher group compared to controls (Figure 1D). The large variation in reaction time within the breacher group could be due to the differences in years of exposure to low levels of blast explosions during the breacher courses (37–40). Note that the source of performance decline may have come from subtle brain network alterations due to blast exposure (41), or could have arisen due to reduced function at the sensory input or neuromuscular output levels (or a combination of all these things). A comprehensive sensory, cognitive, and motor examination of this group (ongoing) will provide these important answers. However, it is also noteworthy that there were overall few CMI deficits in this group of breachers, similar to what we observed in the previously concussed elite-level athletes. Hence, these data suggest the presence of a motor skill reserve reflecting resilient neural movement control networks in these highly trained service members.

Overall, these studies highlight the importance of testing and assessing those affected by blast exposure and mild brain trauma with outcomes that require coordinated brain activity. Assessing motor or cognitive tasks alone do not demonstrate the ability to pick up on the interconnectivity impairments that appear to exist following concussive injuries. Put simply, we believe that multi-domain tasks reveal a failure to communicate, communication here referring to the interaction between different brain areas responsible for rule-based skilled performance.

## What's Going On in There? The Neural Correlates of Cognitive-Motor Integration

We know from behavioral studies that damage to the brain often results in deficits in CMI performance while leaving standard reaching intact. This observation suggests that the neural correlates of CMI differ from those used to produce standard reaching movements. To understand the additional neural control mechanisms involved in movement control using these different levels of gaze/reach dissociation, neuroimaging work in our laboratory has directly compared brain activity in tasks requiring CMI with standard visuomotor mapping. These data provide fundamental information about the neural control of complex movement, and provide insight into what is driving behavioral impairment following diffuse injury from mild head impact.

Many studies suggest that the brain couples eye and arm movements so that they are made to the same spatial location by default (42–44). Within the brain, neural signals associated with the control of the eyes and the arm converge in several regions. For example, posterior parietal cortex regions known to be essential for producing reaching movements encode reaches in an eye-centered frame of reference (45–47). Similarly, the production of arm moments influences activity within brain areas thought to be specific for eye position control (48, 49). The existence of neural circuitry that couples reach to gaze during standard tasks predicts the existence of regions that can inhibit this pairing (50). In a recent functional MRI study, we compared brain activity during standard reaching to activity associated with a CMI task in which saccades were made toward the cued location, but arm movements were made 180° away from that location (51). This task required both decoupling reach from gaze, and incorporating an explicit rule to move the arm away from the cued target location. Patterns of activity within the cuneus region of the occipital cortex, an important sensorimotor hub (52–54), strongly distinguished between the two conditions, predicting whether the eyes and hand were going to move to the same or opposite target locations. Others have observed that damage to the cuneus is a common precursor to the development of optic ataxia (55–58), whereby patients show extremely poor accuracy when reaching for non-foveal targets (59–62). Together, these data suggest that the cuneus is likely crucial for decoupling reach from gaze during CMI tasks. We also observed that like the cuneus, spatial patterns of activity in the medial premotor cortex strongly distinguished between the CMI and standard tasks. Others have reported that when non-human primates learn two different CMI tasks, “context-dependent” cells in this region only fire for one of the two tasks (63). Further, when activity in this region of the brain is suppressed, deficits in switching between rules for different visuomotor associations occur (64). Taken together, these findings suggest that while the cuneus plays a role in allowing us to inhibit the default coupling of the eyes and arm, the medial premotor cortex is involved in incorporating explicit rules into motor plans that determine specifically how these effectors should be dissociated to satisfy the context of a given CMI task.

**Figure 2:**
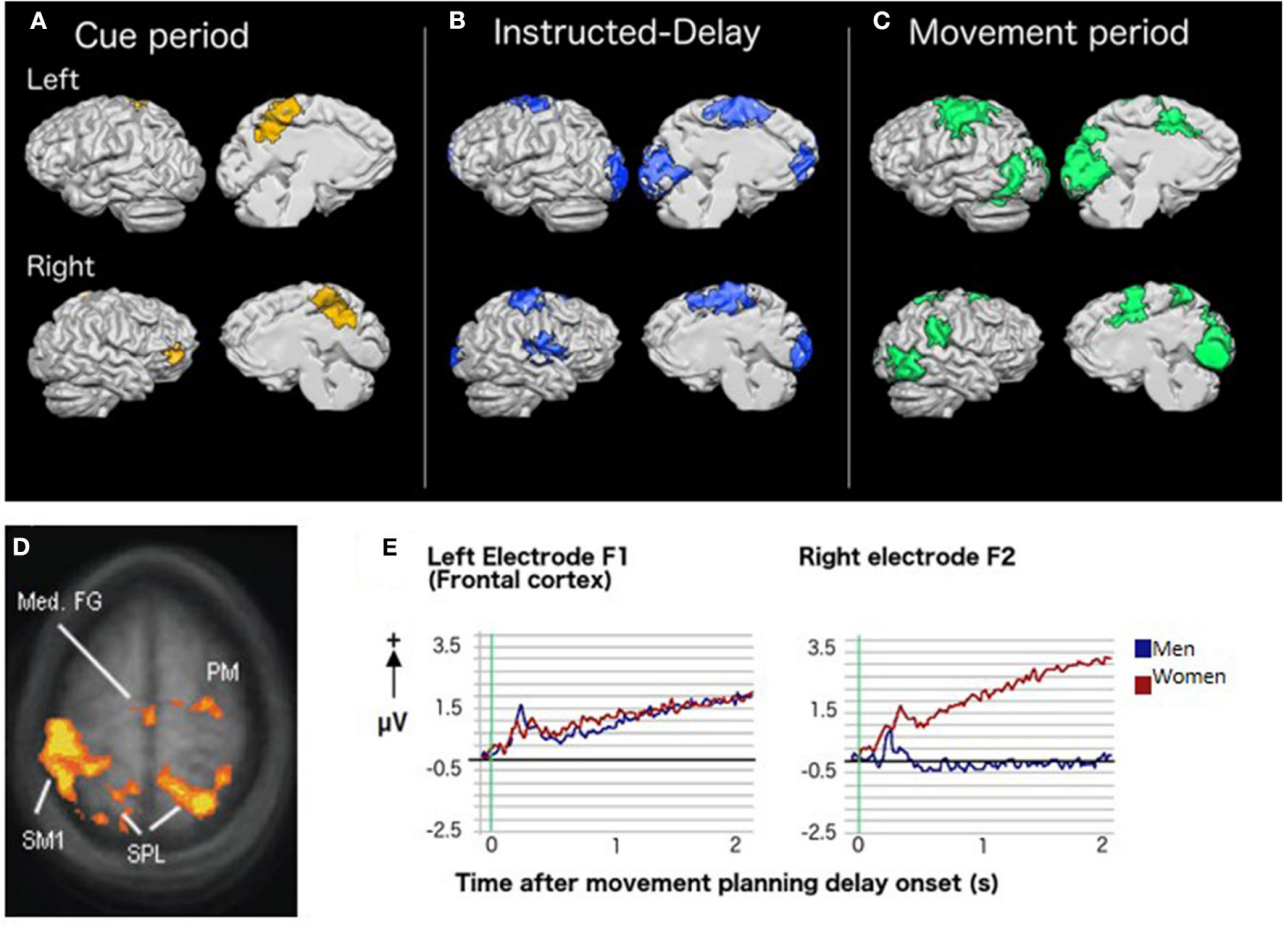
Brain activity and CMI: Results of group whole-brain recursive feature elimination multi-voxel pattern analysis. Colored regions represent voxels in which significant decoding of the standard vs. plane-change visuomotor mapping conditions occurred during **(A)** the cue period (yellow), **(B)** the instructed-delay period (blue), and **(C)** the movement period (green). Regions are shown overlaid on a Talairach-normalized brain from one participant. Within each panel, the left hemisphere is shown at the top and the right hemisphere is shown on the bottom. Lateral surfaces are shown on the left of each panel and medial surfaces on the right [adapted with permission from (65)]. **(D)** Sex-related differences in brain activity during rule-based skill performance. Frontal and parietal cortex regions with significantly higher fMRI BOLD signal in women relative to men during non-standard movement planning [adapted with permission from (66)]. **(E)** EEG slow cortical potentials over left and right frontal cortex during movement planning showing a more bilateral pattern of activity in women than men [adapted with permission from (67)].

CMI tasks also require implicit sensorimotor recalibration. The relationships between visual information, patterns of muscle activity, and proprioceptive feedback are altered. We examined the effect of implicit recalibration in another fMRI study comparing standard hand movements to visual targets on a touch screen in the vertical plane to non-standard ones where the hand moved on a horizontally-placed touch screen (65). Both conditions generated almost completely overlapping activity in the typical motor, somatosensory, premotor, parietal, occipital, and cerebellar regions associated with visually guided reaching, and no differences in the amplitude of task-related activity. However, multi-voxel pattern analyses revealed that within these regions, patterns of activity strongly discriminated between the two tasks. As trials progressed from presentation of a cued target, to a delay period, to motor execution, task discrimination occurred in an increasing number of brain regions, eventually encompassing the majority of regions active in the two tasks (Figures 2A–C). In other words, although both conditions activated similar regions of the brain, the nature of this activity differed in ways that were strongly predictive of which visuomotor mapping was being performed. Taken as a whole, our imaging results in healthy adults demonstrate that each of the additional components required for CMI (i.e., inhibiting the default coupling of reach and gaze, incorporation of task-specific rules, and sensorimotor recalibration) exerts a distinct influence on how movements are accomplished by the brain.

### Sex- and Injury-Related Effects on the Control of Cognitive-Motor Integration

We have observed that both CMI and standard visuomotor tasks evoke a notably more bilateral pattern of activity in premotor and parietal regions in women when compared to men. This phenomenon has been observed using both fMRI (66) and EEG (67). Such bilateral activity in reach-related regions in women might provide protective functional redundancy capable of compensating for decreased activity in either hemisphere, as demonstrated by a study in which one such region (dorsal premotor cortex) was inhibited via transcranial magnetic stimulation (68). Importantly, these sex-related differences are observed even when men and women demonstrate equal skill levels in the tasks (Figures 2D,E). Further, the nature of sex differences can depend upon the specific type of CMI task performed. For example, men had greater lateral sulcus activity than women for CMI tasks in which eye and hand movements were made in opposite directions but not when they moved in the same direction. Others have also observed a pattern of more bilateral brain activity in women relative to men when processing language (69, 70), emotion (71–73), and music (74). Therefore, the phenomenon of relatively bilateral patterns of activity in women is not limited to processes associated with motor control. Different sex-related patterns of brain activity imply that damage to the brain could result in different visuomotor-related deficits in men and women. These observations have important clinical implications and strongly indicate that neurological assessments and rehabilitation must ultimately be customized to best serve the needs of both male and female patients.

Preliminary imaging work has also begun to reveal how a history of concussion affects the neural correlates of CMI. Anatomical images were compared between women who were diagnosed with post-concussion syndrome (PCS) and women with no history of brain trauma (75). When comparing PCS participants to controls, we observed volume decreases in cerebellar regions that serve a variety of diverse roles including cognitive, sensorimotor, and vestibular functions (76). This wide-range of functions could be a factor in the observation that symptoms associated with PCS are quite variable (77). Diffusion-weighted images were also collected, and showed decreased fractional anisotropy (FA) that was associated with poorer CMI performance in several white matter tracts across all study participants (78). Taken with our previous observations that CMI relies upon numerous cortical regions throughout the brain, this finding emphasizes that healthy white matter connections within this network are also crucial for successful cognitive-motor integration.

## Moving Forward, in a Thoughtful Way

As always with science, our research leaves us with more questions than answers. Moving forward, we believe it is important to quantify the effects of various factors that impact one's movement control response to blast exposure and blunt head trauma. Our findings around sex-related differences in the neural control of skill highlight the importance of examining factors such as hormonal influences on injury response, and sex-related vs. gender-related differences in brain network organization. The basic age-related differences we see in our younger vs. working-aged individuals in the studies reviewed here highlights the need to study the interactions between natural aging, skill reserve, and the long-term effects of mild brain injury through longitudinal research. What we are most enthusiastically pursuing at the moment, however, is research into counteracting the effects of compromised brain health through cognitive-motor interventions designed to strengthen the very neural control networks that appear to be affected by concussive injury and neuropathology (79, 80). Such a targeted approach holds promise as an effective means of stabilizing and improving one's functional abilities in the face of injury, allowing for a more enriched daily life.

## Data Availability Statement

The original contributions presented in the study are included in the article/supplementary material, further inquiries can be directed to the corresponding author/s.

## Ethics Statement

The studies involving human participants were reviewed and approved by York University Human Participants Research Committee and the Human Research Ethics Committee of Defence Research and Development Canada. Written informed consent to participate in this study was provided by the participants' legal guardian/next of kin.

## Author Contributions

LS conceptualized the review. LS, DG, MA, and DD conceptualized, wrote, and edited the manuscript. All authors contributed to the article and approved the submitted version.

## Conflict of Interest

The authors declare that the research was conducted in the absence of any commercial or financial relationships that could be construed as a potential conflict of interest.
